# METTL3 Deficiency Aggravates Hepatic Ischemia/Reperfusion Injury in Mice by Activating the MAPK Signaling Pathway

**DOI:** 10.7150/ijms.94177

**Published:** 2024-04-15

**Authors:** Yang Gao, Min Wang, Renyi Qin, Chunle Zhao, Jun Gong

**Affiliations:** 1 Department of Biliary-Pancreatic Surgery, Affiliated Tongji Hospital, Tongji Medical College, Huazhong University of Science and Technology, 1095 Jiefang Ave, Wuhan, 430030, Hubei, China.; 2 Hubei Key Laboratory of Hepato-Pancreato-Biliary Diseases, Affiliated Tongji Hospital, Tongji Medical College, Huazhong University of Science and Technology, 1095 Jiefang Ave, Wuhan, 430030, Hubei, China.

**Keywords:** Hepatic ischemia/reperfusion injury, m^6^A, METTL3, apoptosis, inflammatory response, MAPK.

## Abstract

**Background:** Inflammatory responses, apoptosis, and oxidative stress, are key factors that contribute to hepatic ischemia/reperfusion (I/R) injury, which may lead to the failure of liver surgeries, such as hepatectomy and liver transplantation. The N6-methyladenosine (m^6^A) modification has been implicated in multiple biological processes, and its specific role and mechanism in hepatic I/R injury require further investigation.

**Methods:** Dot blotting analysis was used to profile m^6^A levels in liver tissues at different reperfusion time points in hepatic I/R mouse models. Hepatocyte-specific METTL3 knockdown (HKD) mice were used to determine the function of METTL3 during hepatic I/R. RNA sequencing and western blotting were performed to assess the potential signaling pathways involved with the deficiency of METTL3. Finally, AAV8-TBG-METTL3 was injected through the tail vein to further elucidate the role of METTL3 in hepatic I/R injury.

**Results:** The m^6^A modification levels and the expression of METTL3 were upregulated in mouse livers during hepatic I/R injury. METTL3 deficiency led to an exacerbated inflammatory response and increased cell death during hepatic I/R, whereas overexpression of METTL3 reduced the extent of liver injury. Bioinformatic analysis revealed that the MAPK pathway was significantly enriched in the livers of METTL3-deficient mice. METTL3 protected the liver from I/R injury, possibly by inhibiting the phosphorylation of JNK and ERK, but not P38.

**Conclusions:** METTL3 deficiency aggravates hepatic I/R injury in mice by activating the MAPK signaling pathway. METTL3 may be a potential therapeutic target in hepatic I/R injury.

## Introduction

The liver receives a dual blood supply from the portal vein and the hepatic artery 1, indicating higher sensitivity and susceptibility to ischemia and reperfusion injury. Despite the continuous development of surgical techniques and equipment improving the safety of liver surgery, hepatic ischemia/reperfusion injury (I/R) injury remains a significant cause of postoperative complications and mortality 2. I/R injury is a complex pathological process involving inflammation, cellular apoptosis, and oxidative stress 3, and is regulated by multiple signaling pathways such as the MAPK 4, NF-κB 5 and PI3K/AKT 6, 7 signaling pathways. Despite many studies on the mechanisms involved, few strategies have been translated into clinical practice 8. Thus, an in-depth understanding of hepatic I/R injury may contribute to the development of novel therapeutic approaches to minimize I/R-induced liver damage.

N6-methyladenosine (m^6^A) methylation is one of the most prevalent RNA modifications in eukaryotic cells, mediated by m^6^A writers and is reversible through the action of m^6^A erasers 9. The recognition of such modifications on RNA by m^6^A readers can impact RNA stability and essential biological processes, such as transport and translation 10. m^6^A modification can orchestrate the development, differentiation, and activation of immune cells, consequently affecting the host defense and inflammatory responses to pathogens 11. Dysregulated m^6^A modifications contribute to the pathogenesis of immune-related diseases 12. In addition, many studies have reported the role of m^6^A methylation in programmed cell death including apoptosis, autophagy, pyroptosis, necroptosis, and ferroptosis 13. An increasing understanding of the regulatory mechanisms associated with m^6^A has led to numerous investigations into the role of m^6^A-related molecules in I/R injury in organs such as the heart 14, 15, brain 16, 17, and kidney 18, 19. For instance, m^6^A peaks and genes in the liver were higher in the I/R group than in the sham group using methylated RNA immunoprecipitation and high-throughput sequencing (MeRIP-seq) 20. More importantly, m^6^A demethylase FTO suppressed hepatic I/R injury by demethylating DRP1 mRNA and inhibiting its expression 21.

METTL3 is a well-documented m^6^A writer that collaborates with METTL14, WTAP, and other molecules to introduce m^6^A modifications into RNA 22. METTL3 is expected to be a potential diagnostic biomarker and therapeutic target for various human cancers 22, 23. Recent studies have also revealed that METTL3 is involved in multiple biological processes, such as cell death, through different molecular mechanisms 23. Zhang et al. found that phosphorylated METTL3 could localize to DNA damage sites and contribute to repairing double-strand breaks, thereby protecting cells from cellular death 24. Conversely, Lnc-D63785 m^6^A methylation by METTL3 causes neuronal cell apoptosis 25. METTL3 also plays an important role in inflammatory response. Yang et al showed that METTL3 is upregulated in DSS-induced inflammatory bowel disease, and its overexpression markedly aggravates LPS-induced inflammation in intestinal epithelial cells in mice 26. In rheumatoid arthritis, METTL3 may promote fibroblast-like synoviocyte activation and inflammatory response by activating the NF-κB signaling pathway 27. Nevertheless, the knockdown of METTL3 activated the MAPK signaling pathway to promote pro-inflammatory cytokine expression in LPS-treated osteoblasts 28. In summary, METTL3 is closely associated with the development of apoptosis and inflammation.

Previous studies have confirmed the significance of METTL3 in regulating I/R injuries in organs, including the heart, kidneys, and brain 29-32. However, the role of METTL3 in hepatic I/R injury remains unclear. In this study, we investigated the role of METTL3 in I/R using a mouse model with targeted knockdown and overexpression of METTL3 in hepatocytes. Our results indicated that METTL3 acts as a protective regulator in hepatic I/R injury.

## Materials and Methods

### Animals

Animal experiments were conducted in accordance with the guidelines of the Laboratory Animal Care Evaluation and Accreditation Association and were approved by the Animal Experiment Ethics Committee of Huazhong University of Science and Technology (TJH-202204015). Eight-week-old male C57BL/6 mice were purchased from Gempharmatech Co. Ltd (Jiangsu, China). Mice with the floxed METTL3 allele were generated using CRISPR/Cas9 technology. In brief, single guide RNA sequences were designed on both sides of exon 4 of METTL3, respectively. Then, Cas9 mRNA, sgRNAs, and targeting vector with loxp flanked exon 4 used for homologous recombination, were co-injected into mouse zygotes to generate founder mice. The founder mice were mated with C57BL/6 mice to obtain homozygous mice. METTL3-HKD (METTL3 ^flox/-^; Alb-iCre) mice were generated by mating albumin-iCre and METTL3 ^flox/-^ mice. METTL3-HKD and METTL3-Flox (METTL3 ^flox/flox^) mice from the same litter were obtained by crossing METTL3-HKD and METTL3 ^flox/-^ mice.

### Mouse hepatic I/R model

In this study, a stable mouse model was established using partial (70%) warm liver I/R. The mice were anesthetized and subjected to median laparotomy to expose the liver. Next, the left and middle branches of the intrahepatic portal vein were clamped to block the blood supply. After 1 h of ischemia, the clamp was released for reperfusion. Mice in the sham group underwent the same procedure without the vessels being clamped. Liver and serum samples were collected 3, 6, 12, and 24 h post-reperfusion. At least five mice were harvested at each time point.

### Plasmid construction and transfection

A gene-specific small hairpin RNA sequence was designed and synthesized by Sangon Biotech, and inserted into the pCDH vector. The target sequences were as follows: Mettl3-sh1: GCAAGTATGTTCACTATGAAA; Mettl3-sh2: GCTGCACTTCAGACGAATTAT; Mettl3-sh3: GCCAAGGAACAATCCATTGTT. Transient transfection was carried out in L02 cells with polyethyleneimine (25 kDa, PolyScience, US) according to the manufacturer's protocol. Next, the efficiency of downregulation of the indicated genes was determined by western blotting and qRT-PCR.

### AAV construction and injection

The adeno-associated virus 8 (AAV8) was provided by PackGene Biotech. Co. Ltd (Wuhan, China). Each mouse was injected with 1 × 10^11^ vg virus in 100 μL suspension through the tail vein at their 8 weeks old. The experimental group was injected with AAV8-TBG-METTL3, and the control group was injected with AAV8-GFP. The I/R surgery was performed on AAV virus-treated mice three weeks later. Each group contained at least five mice.

### Total RNA isolation and reverse transcription real-time PCR

Crushed animal liver tissues or cells were used to extract total RNA using Isolator Total RNA extraction reagent (TRIzol, Vazyme, China). And cDNA was reverse-transcribed from total mRNA using HiScript III RT SuperMix for qPCR (Vazyme, China) according to the manufacturer's protocol. qRT-PCR was performed using ChamQ Universal SYBR qPCR Master Mix (Vazyme, China), and the results were analyzed using the Bio-Rad CFX Manager 2.1. Actb was used as an internal reference gene. Primers used are listed in Table S1.

### m^6^A dot blotting analysis

Total RNA was diluted to 400 ng and 200 ng and denatured at 95 °C for 5 min. The RNA dots were placed on and cross-linked with a nylon membrane. The membrane was then washed with PBST for 30 min. Methylene blue (0.02%, Sangon Biotech, China) staining was used to determine the total input RNA content. Another nylon membrane of the coupled RNA was blocked with 5% skim milk and incubated with a specific m^6^A antibody (1:1000, Millipore) at 4 °C overnight, followed by incubation with secondary antibodies at room temperature for 1 h. The obtained dots were visualized with ECL (ThermoFisher, USA) and imaged using the ChemiDoc XRS + system (Bio-Rad Laboratories, USA).

### Alanine transaminase (ALT) and aspartate aminotransferase (AST) measurement

Serum alanine aminotransferase (ALT) and aspartate aminotransferase (AST) levels were measured using the Alanine/Aspartate aminotransferase Assay Kit (Jiancheng Bio, Nanjing, China).

### Isolation of primary hepatocytes

After anesthesia, 50 mL of D-hank's solution (Genview, Beijing, China) was perfused through the portal vein of mice at 37 °C, and 30 mL of D-hank's solution containing type IV collagens (Sigma-Aldrich, USA) was perfused. The infusion was discharged through the inferior vena cava. After stripping the liver, the hepatocytes were suspended in PBS solution at 4 °C and centrifuged in a gradient density Percoll solution (Pharmacia, GE). The liver cells deposited in the bottom layer were planted in petri dishes containing Collagen I (Corning, NY, USA).

### Western blot

The liver tissue was crushed and cleaved in RIPA lysis buffer containing a Protease Inhibitor Cocktail (Boster, Wuhan, China) and PhosSTOP (Roche, USA). The protein samples were separated by 12% or 15% SDS-PAGE and transferred to polyvinylidene fluoride (PVDF) membranes. The membrane was then incubated with specific antibodies for 10 h and with secondary antibodies (Table S2) at room temperature for 1 h before imaging with the ChemiDoc XRS + system (Bio-Rad Laboratories, USA). The signal intensity was quantified using Image J software.

### Histopathology and analysis

Paraffin-embedded mouse liver tissue was cut into 5 μm sections. These sections were stained with hematoxylin and eosin (HE), and histopathological analysis was performed under a light microscope (Mshot, Guangzhou, China). The severity of liver injury was graded according to the Suzuki standard 33. Image Pro Plus software was used to calculate the necrotic area and count TUNEL-positive cells.

### Cell culture and hypoxia/reoxygenation(H/R) modeling

Human embryonic kidney (HEK293T) cells (CTCC, China) and primary hepatocytes were cultured at 37 °C in DMEM (Solarbio Science & Technology Co., China) containing 10% fetal bovine serum (FBS, Cegrogen, Germany), 100 units/mL penicillin, and 100 μg/mL streptomycin. To mimic hepatic I/R injury *in vivo*, L02 cells and primary hepatocytes were placed under hypoxic conditions (1% oxygen) in serum-free DMEM for 6 h and cultured under normal oxygen for another 6 h.

### Statistical analysis

Statistical analysis was performed using SPSS 26.0 (SPSS Inc., USA). All experiments were conducted independently at least three times, and the data are expressed as the mean ± SDs. Unless otherwise indicated, statistical analyses were performed using two-tailed Student's *t*-test. Differences with *P* value < 0.05 were considered statistically significant.

## Results

### m^6^A levels and METTL3 expression were upregulated during liver I/R

**Figure 1:**
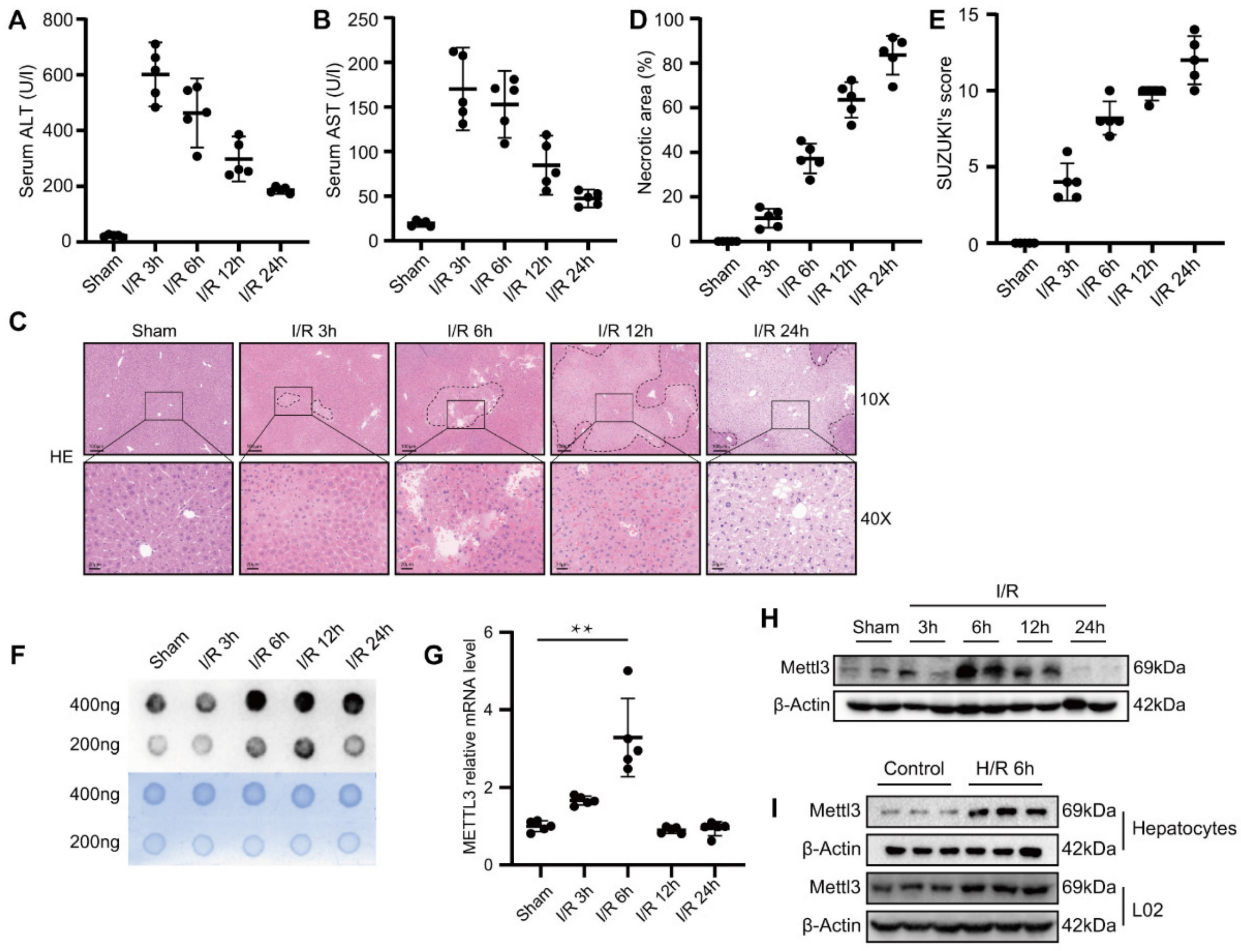
m^6^A** levels and METTL3 expression were upregulated during liver I/R. (A-H)** Wild-type C57BL/6 mice were subjected to sham or 1 h of ischemia and subsequent reperfusion for 3 h, 6 h, 12 h, and 24 h (n = 5 each time point). Relevant indicators were detected. **(A-B)** Serum ALT/AST levels.** (C)** Representative HE staining images of liver sections. **(D)** Necrotic area proportion and **(E)** The Suzuki's scores of liver sections. **(F)** The m^6^A methylation of total RNA was detected by Dot Blot analysis. **(G-H)** The mRNA level **(G)** and protein level **(H)** of METTL3 in the liver tissue harvested from sham and I/R group. **(I)** Western blot analysis of METTL3 expression in primary hepatocytes or L02 cells after 6 h of hypoxia followed by 6 h of reoxygenation.

To monitor changes in m^6^A levels during hepatic I/R, we generated hepatic I/R models using C57BL/6 mice. Compared with the sham group, we found that serum transaminase, ALT, and AST levels were elevated during hepatic I/R **(Fig. 1A-B)**. Consistently, the necrotic area of the liver and Suzuki's score were elevated after I/R **(Fig. 1C-E)**. These results indicate that the hepatic I/R model was successfully constructed and that I/R injury seriously affected liver function in mice. Next, m^6^A dot blot analysis was performed to detect the m^6^A levels of total RNA extracted from the liver tissues of I/R mice. Interestingly, we found that the m^6^A level increased sharply at 6 h and 12 h post-reperfusion, and remained elevated even at 24 h post-reperfusion **(Fig. 1F)**. Knockdown of METLL3, a core component of the methyltransferase complex, results in a significant decrease in global m^6^A levels 34. Thus, we initially determined whether METTL3 levels changed in the liver tissues after I/R injury. As expected, METTL3 mRNA and protein expression level slightly increased at 3 h following hepatic I/R injury and reached the highest level at 6 h post-reperfusion **(Fig. 1G-H)**. In addition, hypoxia and reoxygenation experiments performed on L02 cells and primary mouse hepatocytes demonstrated an increase in the expression of METTL3 during this process **(Fig. 1I)**. Collectively, we speculate that METTL3 may be involved in the regulation of hepatic I/R injury.

### METTL3 deficiency exacerbated liver damage during I/R injury

**Figure 2:**
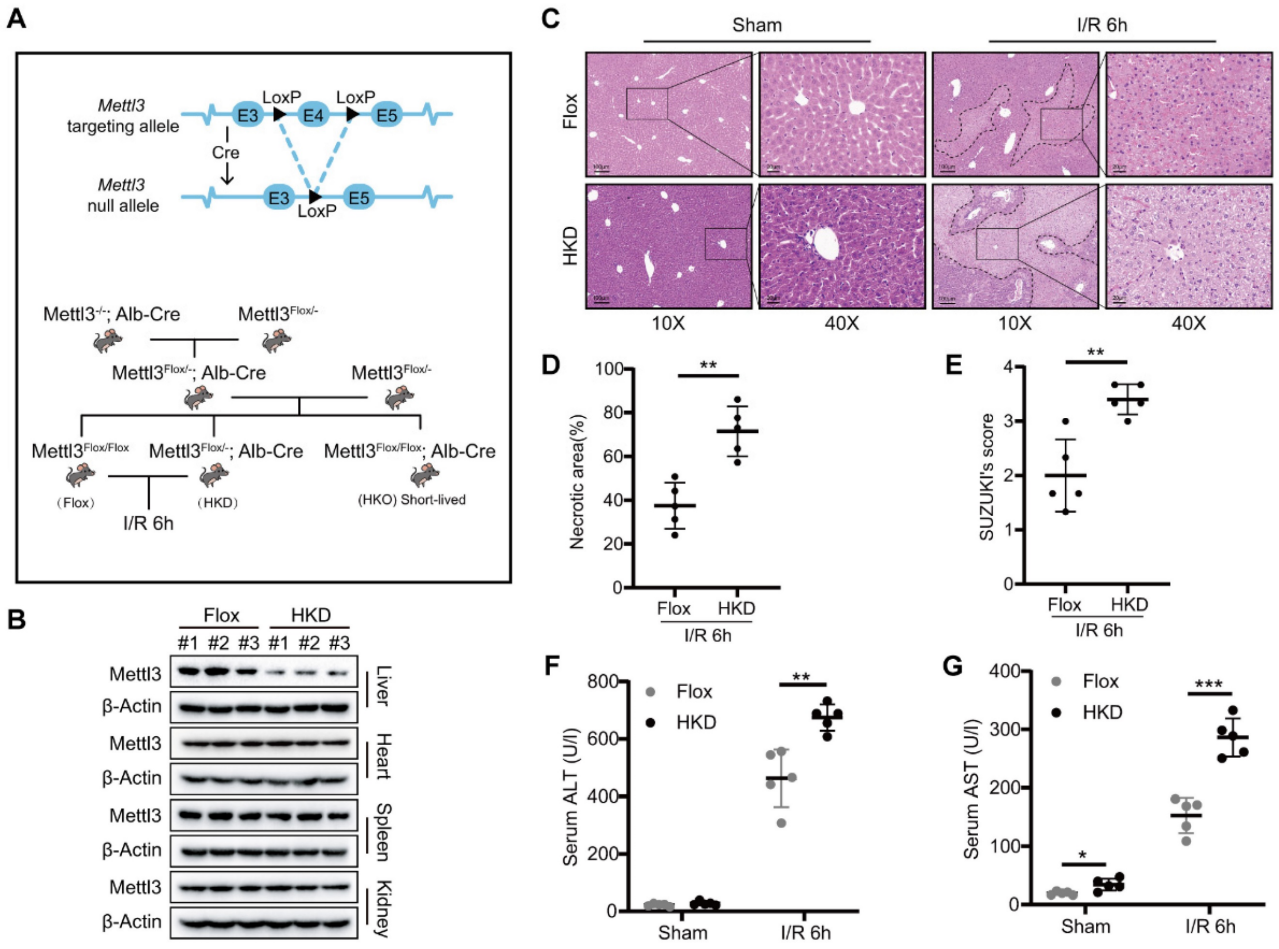
** METTL3 deficiency exacerbated liver damage after I/R injury. (A)** Schematic representation of the generation of METTL3-HKD and METTL3-Flox mice. **(B)** The expression of METTL3 in major organs of HKD mice. **(C-E)** Representative HE staining images** (C)**, necrotic area pro-portion **(D)** and Suzuki' score **(E)** of liver sections, and serum AST/ALT levels **(F-G)** of METTL3-HKD mice and METTL3-Flox mice in the sham group and I/R 6 h group (n = 5 per group).

Next, we generated hepatocyte-specific METTL3 knockout mice (METTL3-HKO) to investigate its role in hepatic I/R injury **(Fig. 2A)**. Unexpectedly, all METTL3-HKO mice died within 6-8 weeks after birth. Thus, we decided to utilize heterozygous mice with reduced expression of METTL3 in the liver (METTL3-HKD) to construct the hepatic I/R model **(Fig. 2A)**. After confirming the specific METTL3 ablation in the liver tissue **(Fig. 2B)**, the genetically engineered mice were subjected to hepatic I/R injury. In consideration of the high expression of both mRNA and protein levels of METTL3 at 6 h post reperfusion, we harvested liver tissues and serum at I/R 6h. Interestingly, HE staining results showed that METTL3-HKD mice exhibited more severe liver damage than control mice at 6 h post-reperfusion. This was evidenced by the larger necrotic area and higher Suzuki score in pathologic staining **(Fig. 2C-E)**. Serum alanine aminotransferase (ALT) and aspartate aminotransferase (AST) levels are important indicators for assessing liver function. Serological tests verified that the serum levels of AST and ALT were significantly higher in METTL3-HKD mice after I/R than in METTL3-Flox mice **(Fig. 2F-G)**. These observations indicated that METTL3 deficiency in hepatocytes exacerbates hepatic I/R-induced liver injury.

### METTL3 deficiency aggravated inflammatory response and cell death during hepatic I/R injury

**Figure 3:**
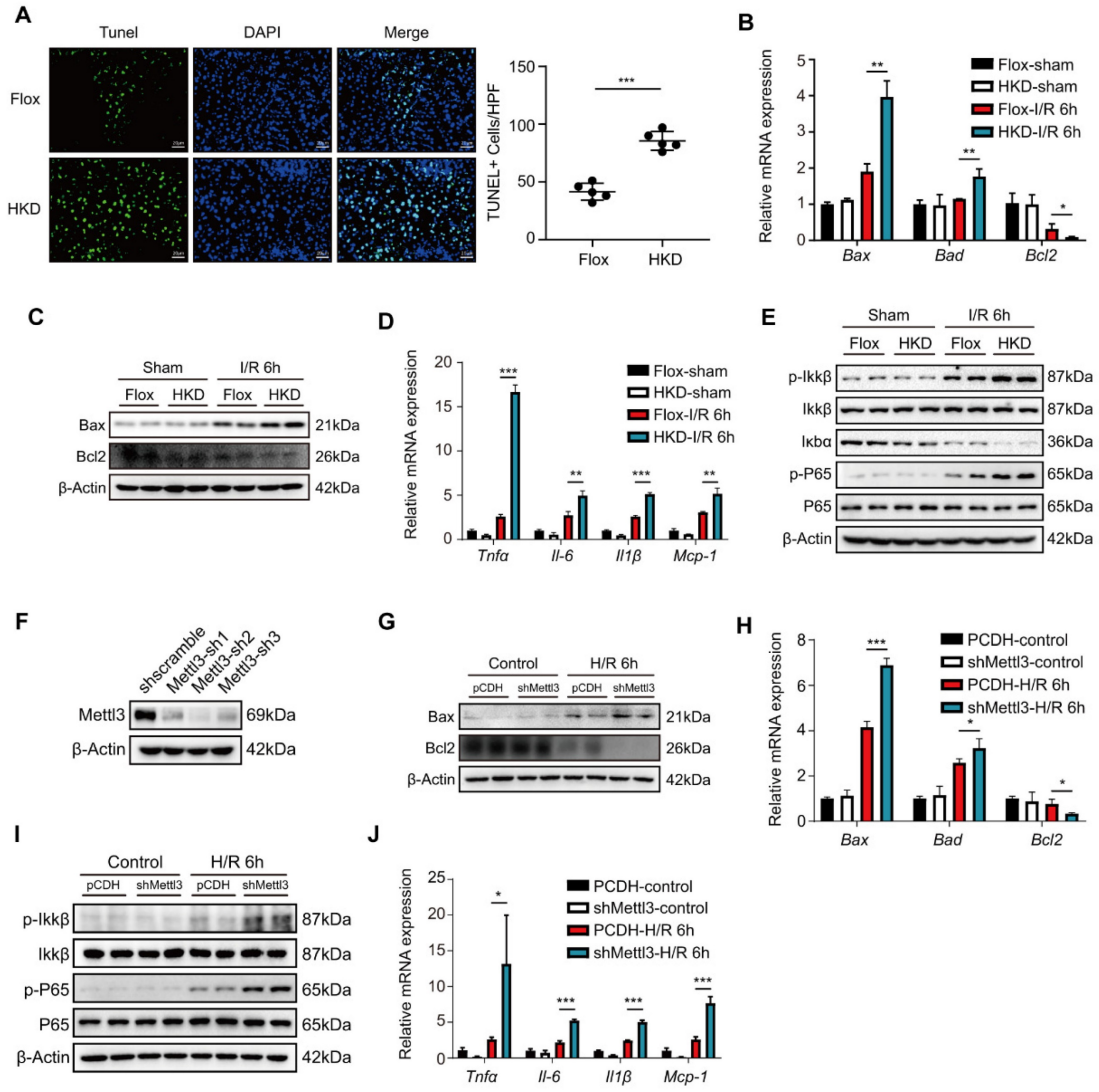
** METTL3 deficiency aggravated inflammatory response and cell death during hepatic I/R injury. (A-E)** Mice were subjected to sham or I/R 6 h injury (n = 5 per group). **(A)** Representative TUNEL staining images and number of TUNEL-positive cells from liver sections of METTL3-HKD and METTL3-Flox mice at 6 h after suffering I/R injury. **(B-C)** The mRNA levels **(B)** and protein levels** (C)** of apoptosis-related factors in the liver. **(D-E)** The mRNA levels of inflammatory factors **(D)** and protein expression of NF-κB signaling pathway **(E)** in the liver. **(F)** L02 cells were transfected with three different shMettl3 plasmids. **(G-J)** Cell death and inflammation-related factors were detected in L02 cells subjected to 6 h of hypoxia and 6 h of reoxygenation after being transfected with shMettl3 or control plasmids.

Apoptosis is a prominent feature and essential step in hepatic I/R injury 35. To investigate the effect of METTL3 on liver cell apoptosis after hepatic I/R, TUNEL staining was performed on liver tissue sections from the I/R mouse models. After I/R, the number of TUNEL-positive cells was significantly higher in the METTL3-HKD mice than in the METTL3-Flox mice **(Fig. 3A)**. In addition, the mRNA levels of the pro-apoptotic factors Bax and Bad were elevated in the METTL3-HKD group, whereas the anti-apoptotic factor Bcl2 was decreased at 6 h post-reperfusion **(Fig. 3B)**. Consistently, the protein expression levels of Bax and Bcl2 showed similar trends (**Fig. 3C**).

As shown in **Fig. 3D**, the levels of pro-inflammatory factors TNF-α, IL-6, and IL-1β, as well as the chemokine MCP-1, were elevated in the livers of METTL3-HKD mice compared with METTL3-Flox mice at 6 h post-reperfusion. Expression of inflammatory factors and chemokines in the liver is associated with the NF-κB pathway. We found that the phosphorylation of IKKβ and p65 was much higher in METTL3-HKD mice than in METTL3-Flox mice 6 h after I/R **(Fig. 3E)**. Given the role of hepatocytes as the major cell type in response to I/R injury 36 and the remarkable upregulation of METTL3 in hepatocytes after H/R challenge **(Fig. 1I)**, we examined the effects of METTL3 on hepatocyte apoptosis and inflammatory responses. We then constructed the METTL3 knockdown cell line through transfecting plasmid with shRNA targeting METTL3 into L02 cells **(Fig. 3F)**. After 6 h of hypoxia followed by 6 h of reoxygenation challenge, we found that METTL3 knockdown elevated apoptosis and inflammatory response-related indicators in hepatocytes **(Fig. 3G-J)**. Taken together, these results suggest that METTL3 deficiency alleviates inflammation and apoptosis in the liver following I/R injury.

### AAV-mediated hepatocyte-specific overexpression of METTL3 attenuated hepatic I/R injury in mice

**Figure 4:**
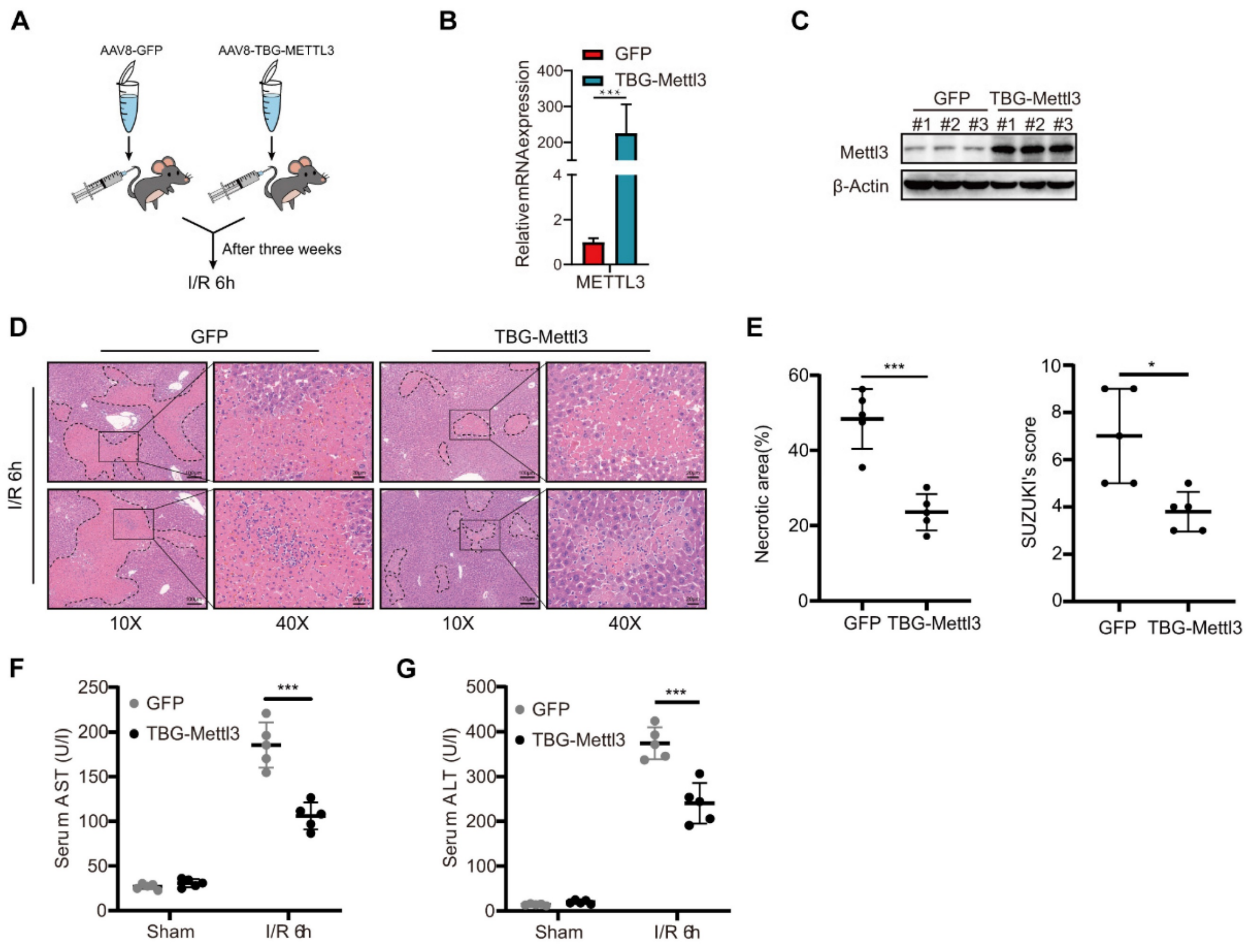
** AAV-mediated hepatocyte-specific METTL3 overexpression attenuated hepatic I/R injury in mice. (A)** Wild type C57BL/6 mice were injected with AAV8-GFP or AAV-TBG-METTL3 through tail vein and then subjected to I/R 6 h injury (n = 5 per group).** (B-C)** Western blot **(C)** and qPCR **(B)** analysis of METTL3 in the liver of mice injected with AAV8-GFP or AAV-TBG-METTL3. **(D-G)** Representative HE staining images **(D)**, necrotic area proportion and Suzuki' score **(E)** of liver sections, and serum AST/ALT levels **(F-G)** of mice injected with AAV8-GFP or AAV-TBG-METTL3 and further subjected to I/R 6 h injury.

Considering that the knockdown of METTL3 in hepatocytes exacerbates severe liver injury after I/R, the overexpression of METTL3 might potentiate its protective role in the liver to some extent. To confirm this hypothesis, C57BL/6 mice were randomly divided and injected with AAV8-TBG-METTL3 or AAV8-GFP **(Fig. 4A)**. qRT-PCR and western blot results demonstrated that viral transfection effectively increased the expression level of METTL3 in the liver **(Fig. 4B-C)**. After the I/R challenge, the HE staining results showed decreased liver necrosis in AAV8-METTL3 mice compared with that in control (AAV8-GFP) mice **(Fig. 4D)**. The corresponding Suzuki's score of histopathology was also reduced in the AAV8-METTL3 mice **(Fig. 4E)**. Furthermore, serum AST and ALT levels were also reduced in AAV8-METTL3 mice compared with AAV8-GFP mice **(Fig. 4F-G)**. These findings suggest that METTL3 overexpression protects against liver damage during hepatic I/R injury.

### Overexpression of METTL3 inhibited inflammation and apoptosis during hepatic I/R injury

**Figure 5:**
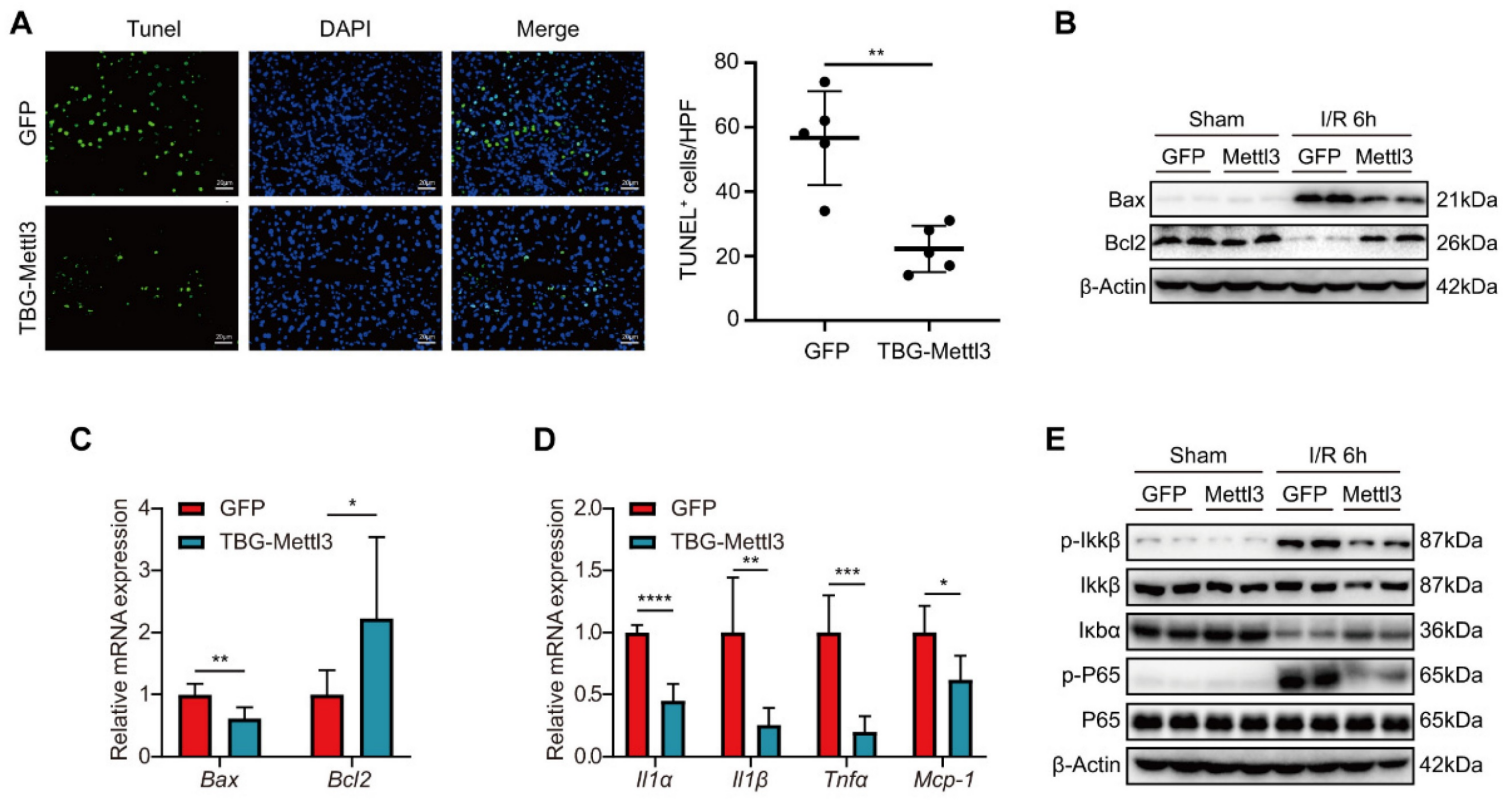
** Overexpression of METTL3 inhibited inflammation and apoptosis during hepatic I/R injury. (A)** Representative TUNEL staining images and numbers of TUNEL-positive cells from liver sections, which were obtained from mice injected with AAV8-GFP or AAV-TBG-METTL3 and further subjected to I/R 6 h injury (n = 5 per group). **(B-C)** The protein levels **(B)** and mRNA levels **(C)** of apoptosis-related factors in the liver of mice injected with AAV8-GFP or AAV-TBG-METTL3 and further subjected to I/R 6 h injury. **(D-E)** The mRNA levels of inflammatory factors **(D)** and protein expressions of NF-κB signaling pathway **(E)** in the liver of mice injected with AAV8-GFP or AAV-TBG-METTL3 and further subjected to I/R 6 h injury.

Next, we investigated whether METTL3 overexpression affected hepatic inflammation and apoptosis and thus exerted protective roles during hepatic I/R injury. Immunofluorescence (IF) staining revealed that the number of TUNEL-positive cells was significantly reduced in liver tissues from AAV8-METTL3 mice relative to control mice at 6 h post-reperfusion **(Fig. 5A)**. Consistently, both protein and mRNA levels of the pro-cell death factor Bax were significantly decreased, whereas the expression levels of the anti-cell death factor Bcl2 were dramatically increased in AAV8-METTL3 mice after I/R challenge **(Fig. 5B-C)**. Moreover, the levels of the inflammatory cytokines IL-1α, IL-1β, tumor necrosis factor TNF-α, and the chemokine MCP-1 were reduced in the liver tissues of AAV8-METTL3 mice **(Fig. 5D)**. Notably, METTL3 overexpression further reduced the protein levels of p-IKKβ and p-P65 but increased IκBα after hepatic I/R injury **(Fig. 5E)**. Collectively, these findings demonstrate that pretreatment with AAV8-METTL3 significantly inhibited hepatic inflammation and apoptosis induced by I/R injury.

### METTL3 participated in hepatic I/R injury by inhibiting the MAPK pathway

**Figure 6:**
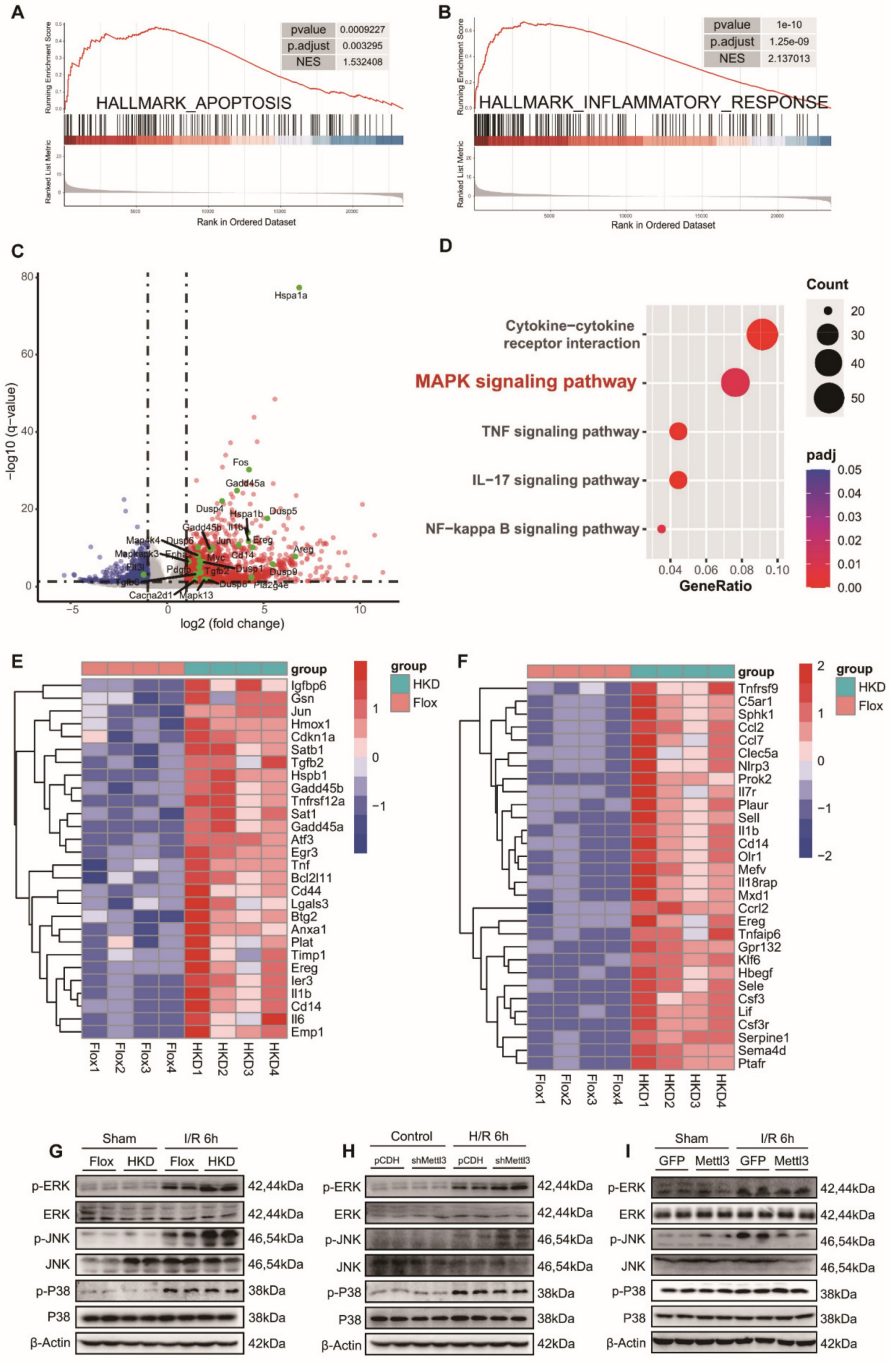
** METTL3 participated in hepatic I/R injury by inhibiting the MAPK pathway. (A-B)** GSEA analysis of the biological processes related to apoptosis **(A)** and inflammatory response **(B)**. **(C)** Volcano plots showing the significantly differential genes between the METTL3-HKD and METTL3-Flox mice subjected to I/R 6 h injury. MAPK signaling pathway-related genes are high-lighted in green. **(D)** Major biological pathways determined by KEGG enrichment analysis of RNA-seq of liver tissues from METTL3-HKD and METTL3-Flox mice subjected to I/R 6 h injury. **(E-F)** Heatmap generated based on the expression of apoptosis-related genes **(E)** and inflammation-related genes **(F)** in the livers from METTL3-HKD and METTL3-Flox mice detected by RNA-seq analyses. **(G-I)** Western blot analyses of the total and phosphorylated protein levels of ERK, JNK, and P38 in liver and L02 cells after I/R or H/R insult.

To gain further insight into the molecular events underlying the exacerbation of liver injury caused by METTL3 deficiency, we conducted RNA-seq analysis using liver tissues from METTL3-HKD mice and METTL3-Flox mice that were ischemic for 1 h followed by reperfusion for 6 h. Consistent with our previous findings, the Gene Set Enrichment Analysis (GSEA) results showed a significant enrichment of “HALLMARK APOPTOSIS” and “HALLMARK INFLAMMATORY RESPONSE” items **(Fig. 6A-B)**. To identify differentially expressed genes (DEGs), an adj *P* value < 0.05 and a |Log2(Fold Change)| ≥1 were established as thresholds. Volcano mapping showed that 1117 genes were upregulated and 321 genes were downregulated in METTL3-HKD mice compared to those in METTL3-Flox mice **(Fig. 6C and Table S3)**. KEGG enrichment analysis based on DEGs highlighted the mitogen-activated protein kinase (MAPK) signaling pathway, cytokine-cytokine receptor interaction, and other significantly enriched signaling mechanisms during hepatic I/R injury **(Fig. 6D)**. The cluster diagram shows the details of the top differentially expressed inflammation-related and apoptosis-related genes **(Fig. 6E-F)**. Next, we assessed the effect of METTL3 on MAPK pathway activation following hepatic I/R injury. The results showed that all three kinases were activated after I/R injury, whereas METTL3 deficiency further increased the levels of p-ERK and p-JNK, and had little effect on p-P38 expression **(Fig. 6G)**. Activation of ERK and JNK was also observed in METTL3-knockdown L-02 cells after H/R insult **(Fig. 6H)**. Moreover, the opposite protein expression profiles were observed in AAV8-treated mice **(Fig. 6I)**. These findings support the notion that METTL3 attenuates hepatic I/R injury by modulating activation of the MAPK signaling pathway.

## Discussion

Currently, the prevention and treatment of I/R injury after liver transplantation and hepatectomy remain limited, lacking effective targeted drugs 37. The identification and characterization of novel regulatory factors may elucidate pathogenesis and provide therapeutic targets for hepatic I/R injury. Here, we found that the expression of METTL3 significantly increased after hepatic I/R, peaking at 6 h post-reperfusion. Further studies revealed that the deletion of METTL3 in hepatocytes resulted in a significant aggravation of liver injury. In contrast, the overexpression of METTL3 using AAV8 effectively attenuated the degree of liver injury after I/R. Our results suggested that METTL3 is a protective factor against liver injury.

Studies have shown that m^6^A plays a crucial role in the physiological functions of liver cells, as well as in the pathogenesis of liver diseases and cancers 38. METTL3, an important member of m^6^A writers, regulates inflammatory diseases by modulating the function of inflammatory immune cells 39.

However, there have been limited studies on the relationship between METTL3 and hepatic I/R injury, a condition in which apoptosis and inflammation are essential therapeutic mechanisms 40. To explore the role of METTL3 in hepatic I/R injury, we developed METTL3-HKD mice and injected AVV-TBG-METTL3 into the tail veins of wild-type mice to enhance the expression of METTL3 in the liver. Two sets of mice were utilized to construct I/R models, and related indexes were detected, which included changes in serum liver enzymes, inflammatory factors, and apoptosis factors. Interestingly, we found that hepato-specific knockdown of METTL3 resulted in increased apoptosis damage and inflammation levels, whereas increasing METTL3 expression significantly reduced inflammation and apoptosis levels. Thus, our study suggests that METTL3 plays a role in the regulation of inflammation and apoptosis in this injury, potentially by inhibiting activation of the MAPK pathway to influence inflammation levels. However, the specific targets of METTL3 require further investigation, which is a limitation of our study.

Xiang et al. reported an association between METTL3 and post-reperfusion injury in rat liver transplantation 41. The authors concluded that METTL3 could attenuate I/R injury in transplanted livers, which is consistent with our findings. However, they observed reduced levels of m^6^A modification and downregulation of METTL3 expression in liver grafts, which contradicts our results. We speculated that these discrepancies might be attributable to differences between rat and mouse species, as well as differences in modeling methods. For instance, the immunological response in the liver transplantation model would be more complex than in the I/R model, because the transplanted liver needs to receive reperfusion from the new host. More importantly, we found that METTL3 was upregulated after hepatic I/R injury, whereas its deficiency exacerbated the liver injury. These contradictory results could be interpreted as an intrinsic self-protective mechanism triggered by the organism in response to I/R injury, including the upregulation of METTL3 expression, which was only partly inhibited after reperfusion.

The MAPK signaling pathway has been reported to be involved in the regulation of hepatic I/R injury 42, 43. By analyzing the RNA-seq data of liver tissues from METTL3-HKD mice and METTL3-Flox mice, we found an enrichment of the MAPK signaling pathway. METTL3 deficiency resulted in further activation of the MAPK signaling pathway in I/R injury, which was evidenced by the increased phosphorylation of ERK and JNK. Activated ERK has been reported to be associated with cell proliferation and could exacerbate I/R injury, which might be attributed to the infiltration of neutrophils and cell death 44.

In conclusion, our study demonstrates the role of METTL3 molecules in hepatic I/R injury. But there are still some limitations. More experiments are needed to explain how METTL3 affects the MAPK signaling pathway in order to pinpoint effective drug targets.

## Supplementary Material

Supplementary tables 1-2.

Supplementary table 3.

## Abbreviations

AAV: Adeno-associated virus
ALT: Alanine aminotransferase
AST: Aspartate aminotransferase
HE: Hematoxylin and Eosin
HKD: Hepatocyte-specific knockdown
IF: Immunofluorescence
I/R: Ischemia/Reperfusion
MAPK: Mitogen-activated protein kinase
M^6^A: N6-methyladenosine
NF-κB: Nuclear factor kappa-B
