# Supplementary material for: METTL3 Deficiency Aggravates Hepatic Ischemia/Reperfusion Injury in Mice by Activating the MAPK Signaling Pathway
Source: Int J Med Sci. 2024 Apr 15;21(6):1037–48. doi: 10.7150/ijms.94177 (PMC11103385; doi:10.7150/ijms.94177)
Supplement: Supplementary file 1 — Supplementary tables 1-2. [file ijmsv21p1037s1.pdf]

Table S1. Primers for quantitative PCR

Primer set	Primers	Sequence (5'-3')	Product size (bp)
m-*Mettl3*	Forward	5'-CTGGGCACTTGGATTTAAGGAA-3'	173
	Reverse	5'-TGAGAGGTGGTGTAGCAACTT-3'	
h-*Mettl3*	Forward	5'-TTGTCTCCAACCTTCCGTAGT-3'	145
	Reverse	5'-CCAGATCAGAGAGGTGGTGTAG-3'	
m-*Bax*	Forward	5'-TGAGCGAGTGTCTCCGGCGAAT-3'	213
	Reverse	5'-GCACTTTAGTGCACAGGGCCTTG-3'	
h-*Bax*	Forward	5'-CCCGAGAGGTCTTTTTCCGAG-3'	155
	Reverse	5'-CCAGCCCATGATGGTTCTGAT-3'	
m-*Bad*	Forward	5'-CCAGAGTTTGAGCCGAGTGAGCA-3'	173
	Reverse	5'-ATAGCCCCTGCGCCTCCATGAT-3'	
h-*Bad*	Forward	5'-CCCAGAGTTTGAGCCGAGTG-3'	249
	Reverse	5'-CCCATCCCTTCGTCGTCCT-3'	
m-*Bcl2*	Forward	5'-TGGTGGACAACATCGCCCTGTG-3'	118
	Reverse	5'-GGTCGCATGCTGGGGCCATATA-3'	
h-*Bcl2*	Forward	5'-GGTGGGGTCATGTGTGTGG-3'	89
	Reverse	5'-CGGTTCAGGTACTCAGTCATCC-3'	
m-*Tnfα*	Forward	5'-CATCTTCTCAAAATTCGAGTGACAA-3'	175
	Reverse	5'-TGGGAGTAGACAAGGTACAACCC -3'	
h-*Tnfα*	Forward	5'-TGGCGTGGAGCTGAGAGATA-3'	176
	Reverse	5'-TGATGGCAGAGAGGAGGTTG-3'	
m-*Il6*	Forward	5'-AGTTGCCTTCTTGGGACTGA-3'	159
	Reverse	5'-TCCACGATTTCCCAGAGAAC -3'	
h-*Il6*	Forward	5'-GAGTAGTGAGGAACAAGCCAGA-3'	192
	Reverse	5'-AAGCTGCGCAGAATGAGATGA-3'	
m-*Il1β*	Forward	5'-CCGTGGACCTTCCAGGATGA-3'	117
	Reverse	5'-GGGAACGTCACACACCAGCA-3'	
h-*Il1β*	Forward	5'-GCTGGAGAGTGTAGATCCCAAA-3'	139

	Reverse	5'-TGCTTGAGAGGTGCTGATGT-3'	
m-*Mcp-1*	Forward	5'-TGGCTCAGCCAGATGCAGT-3'	81
	Reverse	5'-CCAGCCTACTCATTGGGATCA -3'	
h-*Mcp-1*	Forward	5'-ATAGCAGCCACCTTCATTCCC-3'	154
	Reverse	5'-CAGCTTCTTTGGGACACTTGC-3'	

Table S2. Information for antibodies used in this study

Protein	Assay	Catalog number	Company
METTL3	WB	15073	Proteintech
β-Actin	WB	AC026	ABclonal
Bax	WB	60267	Proteintech
Bcl2	WB	3498T	Cell Signaling Technology
p-IKKβ	WB	AP1237	ABclonal
IKKβ	WB	A0714	ABclonal
IκBα	WB	A11397	ABclonal
p-P65	WB	AP0446	Abclonal
P65	WB	10745	Proteintech
p-ERK	WB	4370T	Cell Signaling Technology
ERK	WB	11257	Proteintech
p-JNK	WB	4668S	Cell Signaling Technology
JNK	WB	17572	Proteintech
p-P38	WB	9216S	Cell Signaling Technology
P38	WB	170099	Abcam
